# Supplementary material for: Incidence and risk factors for medical care interruption in people living with HIV in a French provincial city
Source: PLoS One. 2020 Oct 15;15(10):e0240417. doi: 10.1371/journal.pone.0240417 (PMC7561150; doi:10.1371/journal.pone.0240417)
Supplement: Attachment — Submitted filename: Response to Reviewers.docx [file pone.0240417.s001.docx]

**Response to the academic editor and reviewers’ comments on the paper**

**PONE-D-20-20346**

We thank the editor for giving us the opportunity to present our study, as well as the reviewer for his(her) comments.

**Journal Requirements:**

**The revised manuscript was edited to meet PLOS ONE’s style requirements.**

**Here is our updated Competing Interests statement as required:**

**“**I have read the journal's policy and the authors of this manuscript have the following competing interests: JJP reports grants from ViiV Healthcare and MSD, and personal fees from Gilead, ViiV Healthcare and MSD outside the present work. KC has served as a speaker and as a consultant for Gilead outside the present work. YY has served as a speaker and as a consultant for Abbott, Bristol-Myers Squibb, Gilead, MSD, Roche, Tibotec and ViiV Healthcare outside the present work. RV received travel grants from Gilead, Merck and ViiVHealthCare outside the present work. ALF, PF and EP have no conflicts of interest to declare. This does not alter our adherence to PLOS ONE policies on sharing data and materials.”

**Editor's comments:**

As the editor required, the revised manuscript was edited for proper English language, grammar, punctuation, spelling, and overall style by one or more of the highly qualified native English speaking editors at American Journal Experts.

**Reviewers' comments:**

Reviewer #1: The study titled "Incidence and risk factors for medical care interruption in people living with HIV in a French provincial city" PONE-D-20-20346 is a valuable work. Continuity of medical care is very important, especially in PLWH because of regular antiretroviral treatment and immunodeficiency - they are special patients. That is why the topic about medical care interruption among PLWH is very interesting.
Abstract contains the most important issues. The introduction is thoughtful and leads to the main topic. The research methods are clear and the patients cohort is quite large. Statistical analyses are written well. The results are detailed and the tables are in proper format. The discussion the limitations of the study are included as well. The discussion compares studies not only from France but also from other countries, which is additional value of this study.
I would suggest to check the paper by native speaker - there are some small English language mistakes.

A native-English speaking editors at American Journal Experts reviewed this version of the manuscript. A certificate issued on September 18, 2020 may be verified on the AJE website using the verification code 2E8D-FB26-C6A6-1AE2-8F86.
